# Emotions and ensuing motor performance are altered by regulating breathing frequency: Implications for emotion regulation and sport performance

**DOI:** 10.3389/fpsyg.2022.963711

**Published:** 2022-10-06

**Authors:** Taylor L. Buchanan, Christopher M. Janelle

**Affiliations:** ^1^Center for Exercise Medicine, Division of Gerontology, Geriatrics, and Palliative Care, College of Medicine, University of Alabama at Birmingham, Birmingham, AL, United States; ^2^Performance Psychology Laboratory, Center for Exercise Science, Department of Applied Physiology and Kinesiology, College of Health and Human Performance, University of Florida, Gainesville, FL, United States

**Keywords:** breathing rate, emotion regulation, motor performance, heart rate variability, arousal

## Abstract

Breathing interventions have been shown to improve sport performance. Although evidence exists to support the role of perceived arousal as a critical underlying mechanism of breathing interventions, methodological differences in the literature preclude clear understanding of potential contributing factors to the effectiveness of such interventions. Under neutral contexts, we have demonstrated attention, dyspnea, and hindrance may need to be considered as mediators of how breathing frequency affects motor performance. We sought to extend our previous findings to determine how breathing frequency affects motor performance under varying emotional conditions. Participants (*N* = 35, *Mage* = 21.68, *SD* = 2.96; 20 females) performed slow, normal, and fast metronome-paced breathing while viewing pleasant and unpleasant stimuli prior to executing a pinch grip task. Performance was assessed *via* reaction time (RT), variability (V) and error (AE). Assessment of indices of perceived arousal included measuring heart rate variability (HRV) and visual analog scale responses. Visual analog scales were also used to assess attention, dyspnea, and hindrance. Repeated measures ANOVAs showed slow breathing increased RT and HRV compared to normal and fast breathing under emotional conditions (all *p*’s < 0.05). Hierarchical multiple regression models revealed that decreased breathing frequency predicted increases in RT (*β* = −0.25, *p* < 0.05) under pleasant conditions, while predicting increases in HRV for unpleasant conditions (*β* = −0.45, *p* < 0.001). Increases in dyspnea (*β* = 0.29, *p* < 0.05) and hindrance (*β* = 0.35, *p* < 0.01) predicted increases in RT under pleasant conditions, while only increases in hindrance predicted increases in RT under unpleasant conditions (*β* = 0.41, *p* < 0.01). Decreases in breathing frequency predicted increases in HRV under unpleasant conditions (*β* = −0.45, *p* < 0.001). Overall, our findings suggest under varying emotional contexts breathing frequency differentially affects movement, potentially mediated by factors other than perceived arousal. In addition, these results inform the use of breath regulation as an antecedent emotion regulation strategy.

## Introduction

On a daily basis, emotions are experienced in response to the surrounding environment and the stimuli that inform and enrich the human experience (Jacobsen, 2018). Characteristics of environmental stimuli elicit responses that are shaped by the emotional reactions experienced in a specific context or situation (Gross, 2015). In performance contexts, emotions may facilitate or inhibit movement, including attempts to regulate those same emotions. Highly arousing performance environments such as competitive sports (Lazarus, 2000), surgical training (Panayiotou Charalambous, 2017), firearm control (Sade et al., 1990), and driving (Montoro et al., 2018) can induce maladaptive emotional states and result in injuries caused by errors in movement quality (Shanafelt et al., 2010; Zhu and Srinivasan, 2011; Hsu et al., 2017; Rice and Schroeder, 2019). Such contexts can also elicit peak performance at precisely the moment it matters most when emotion regulation – either implicitly or explicitly exerted – organizes psychophysiological conditions that permit optimal movement planning and execution.

Breathing interventions, particularly those in which breathing frequency is manipulated, offer promising, low cost, unobtrusive approaches to beneficially impact the autonomic nervous system as well as perceived emotional states (Schuman and Killian, 2019). Slow breathing frequency has been associated with reductions in perceived negative affect (Ma et al., 2017), while fast breathing is associated with increases in perceived negative affect, including discrete emotions such as anger and fear (Philippot et al., 2002; Ali et al., 2018). However, even “negative” emotional experiences can be of benefit in performance contexts, eliciting increases in speed and power that may be ideal for performance of many sport tasks. The Temporal Influence Model of Emotion Regulation (TIMER; Beatty and Janelle, 2020) provides a framework for contextualizing sports characteristics and the importance of timing with regard to the implementation of regulation strategies. Under TIMER, breath regulation interventions are categorized as response modulation strategies and can be implemented at pre-performance, active performance, and post-performance timepoints (Beatty and Janelle, 2020). Breathing slowly before a free throw shot in basketball, during a baseball pitch, and after tennis point are all reasonable implementation periods for breath regulation. The regulatory effects of slow and fast breathing frequency on emotion alone translate well to performance contexts where breathing rate can be purposefully altered prior to or during performance.

Breathing interventions have been shown to improve sport performance. More specifically, reduced breathing frequency has been shown to improve dance technique in professional dancers (Raymond et al., 2005), and dribbling, shooting, and passing motor skills in basketball players (Paul and Garg, 2012). Additionally, biathletes sync their respiratory rate to shoot targets, and runners and swimmers sync their respiration with movement cadence (McDermott et al., 2003; Sattlecker and Lindinger, 2007; Leahy et al., 2019). While evidence exists to support the notion that controlled breathing frequency and depth can influence motor behavior, the mechanisms underlying these effects remain unclear.

Many sports require planning-dependent movements, and it is therefore essential to select an appropriate strategy within available time-constraints. In order to study the impact of emotions or emotion regulation strategies on motor planning in a controlled manner, a pinch-grip task provides an ideally suited, simple yet elegant option. Among other advantages, we have used the pinch grip task in past research to study foundational questions of how emotions affect motor planning (Coombes et al., 2009). Most recently, we demonstrated that motor planning of a pinch grip task was predictably modified by implementing breathing in the pre-performance time point (Buchanan and Janelle, 2021). The evidence generated in these previous projects provide a firm foundation upon which to understand ensuing manipulations of emotional reactivity, regulation, and performance.

Breathing interventions can be customized to manipulate respiratory parameters that in turn alter the physiological mechanisms that contribute to performance (Song and Lehrer, 2003). In athletic populations specifically, it is postulated that HRV changes as an indirect by-product of manipulating breathing frequency to improve performance outcomes (Jimenez Morgan and Molina Mora, 2017). Breathing rate and depth directly influence physiological arousal, which underscores mechanistic explanations for how and why breathing interventions are effective in performance settings (Tipton et al., 2017; Mather and Thayer, 2018). Attention allocation is another potential mechanism underlying the efficacy and efficiency of breathing interventions in performance contexts. Performance contexts require constant processing of external emotional stimuli, resulting from top-down (goal-directed) and bottom-up (stimulus-driven) attentional systems (Eysenck et al., 2007). Breathing frequency interventions direct attention away from emotional stimuli to one’s breath, potentially enhancing the interventions’ effects on emotions. Depending on the specific breathing rate and motor demands of the performance context, the effect of breathing interventions may be facilitative or inhibitive (Koole, 2010).

In addition to addressing the influence of attention, recently published work has indicated that dyspnea (shortness of breath) and hindrance (a sense of something being detrimental to task performance) play a role in the association between breathing and performance, with fast breathing increasing perceived dyspnea and hindrance to a motor performance task(Buchanan and Janelle, 2021). Our prior work identified that fast and slow breathing is associated with perceived and objective arousal patterns that impact reaction time, a necessary parameter of motor performance (Buchanan and Janelle, 2021). Under varying emotional contexts, these factors, may affect the influence of breathing rate on performance parameters. Unpleasant emotional contexts speed up reaction time, suggesting such contexts prime the motor system (Coombes et al., 2009). The importance of understanding how breathing impacts performance under emotional contexts is critical to furthering an understanding of breath regulation as an emotion regulation strategy in performance contexts such as sport. Yet, the extant database addressing the interaction of breathing, emotion, and performance remains limited.

The purpose of this study was to determine whether and how breathing frequency influences performance of a rapid force pulse pinch-grip movement under varying emotional conditions. We hypothesized fast breathing rates will increase error and maintain reaction time compared to slow and normal breathing rates under pleasant and unpleasant emotional conditions. Additionally, we hypothesized breathing frequency would predict changes in perceived arousal under pleasant and unpleasant emotional conditions. We also expected increased attention to breathing would predict changes in variability and error under pleasant and unpleasant emotional conditions. Based on increases in dyspnea being related to increases in attentional demand, we therefore hypothesized that participants would feel greater levels of dyspnea and hindrance.

## Materials and methods

### Participants

Thirty-five healthy right-handed adults (age *M* = 21.68, *SD* = 2.96; 20 females) were recruited from various courses in the Department of Applied Physiology and Kinesiology at the University of Florida for participation in the study. Extra course credit (<2.5% of final grade) was provided in return for participation. All participants provided written informed consent approved by the University’s Institutional Review Board. Participants were advised to follow a normal sleep routine including refraining from intense physical activity, and drinking alcohol the day before the experiment. In addition, participants were instructed to refrain from eating meals and drinking caffeinated beverages. On the day of the experiment, participants were further directed to wear comfortable clothing and use the restroom prior to arrival. All pre-test instructions were given based on advisement of how each factor may impact HRV (Laborde et al., 2017).

Throughout recruitment, individuals were excluded if they self-reported any current movement disorders or injuries that could alter their motor behavior, respiratory or cardiovascular disorders that could alter breathing behaviors, psychological disorders that could alter their emotional behavior, or hearing or vision problems that may impact comprehension and performance of experimental tasks. Recruited individuals scored within normal ranges on the Depression, Anxiety, Stress Scale (DASS-21; Lovibond and Lovibond, 1995), and did not report any contraindications on the Physical Activity Readiness Questionnaire (PAR-Q; Thomas et al., 1992). Characteristics of participants are presented in Table 1.

**Table 1:** Depression, anxiety, stress, scale, and age values *M* (*SD*) values for participants included in the statistical analyses.

Variable	Participants (*N* = 35)
Age (years)	21.68 (2.96)
DASS-Total	13.33 (11.55)
DASS-D	3.64 (4.17)
DASS-A	2.97 (3.89)
DASS-S	6.72 (5.95)

### Questionnaires

When participants first arrived at the laboratory, they filled out the Depression Anxiety Stress Scale (DASS), which is a 21-question self-reported questionnaire measuring the emotional states of depression, anxiety, and stress, accordingly. Each question is rated on a 4-point scale with 0 as the lowest score and 3 as the highest score for each of the items that are presented. We implemented the DASS as a validated reliable tool for reporting participants’ emotional states. Although not used for diagnostic purposes, the scale correlates well with the Beck Depression and Anxiety Inventories (BDI) and (BAI) inventories. The reliability scores of the DASS-21 scales are.88 for Depression, 0.82 for Anxiety, 0.90 for Stress, and.93 for the Total scale. Validity is similar to that of the original DASS scale (Lovibond and Lovibond, 1995; Table 1).

Following each condition participants completed the Affect Grid. The Affect Grid quantifies perceived valence and arousal levels using a 9 × 9 grid, with coefficient alpha estimates of reliability of.87 for PA and.79 for NA (Russell et al., 1989). The horizontal axis represents valence: 1 (unpleasant) to 9 (pleasant). The vertical axis represents arousal: 1 (low) to 9 (high). The Affect Grid was selected as it simultaneously measures the dimensional properties of emotion. Following experimental conditions, participants rated overall valence and arousal experienced during breathing and the pinch task (Table 2).

**Table 2:** Ratings of perceived emotion, attention, dyspnea, and hindrance for all participants across each breathing condition.

Variable	Conditions			
	Baseline	Slow	Normal	Fast
Arousal	5.23 (1.65)	4.03 (1.64)	3.89 (1.93)	5.00 (2.07)
Valence	5.77 (1.31)	5.43 (1.69)	5.14 (1.78)	4.32 (2.07)
Attention	8.33 (1.67)	3.38 (2.14)	3.89 (2.33)	2.92 (2.15)
Dyspnea	1.71 (1.75)	3.49 (2.21)	3.73 (2.25)	5.13 (2.62)
Hindrance	2.24 (2.56)	5.71 (2.65)	5.65 (2.49)	6.77 (2.22)

Means (*M*) and standard deviations (*SD*) are presented for each dependent variable: Arousal and Valence ratings were acquired using Likert Scales (1–9), and Attention, Dyspnea and Hindrance using Visual Analog Scales (cm) were on a scale of 1–10.

Visual analog scales for attention, hindrance, and dyspnea (shortness of breath) experienced were given following each breathing condition. Using a visual analog scale (VAS), allowed detection of small changes within individuals’ emotional state and attention. Participants were asked to mark a vertical dash on a horizontal line based on how they felt breathing impacted performance on the pinch task, numbered 0 to 10. For the VAS of attention, 0 indexed attention to the breathing task and 10 indexed attention to the pinch task. For the VAS of hindrance, 0 indexed breathing rate did not hinder performance of pinch task and 10 indexed breathing rate did hinder performance on pinch task. For the VAS of dyspnea, 0 indexed no noticeable or unpleasant shortness of breath caused by breathing rate and 10 indexed maximal imaginable intensity and unpleasantness caused by breathing rate. Validity of visual analog scales are well established for breathing, emotion, and performance (Gift, 1989; Ahearn, 1997; Turner et al., 2008). Scales were implemented to determine if individual’s expectations of performance were associated with objective outcomes of performance (Table 2).

### Heart rate variability

HRV is the complexity of the timing between heart beats and is indicative of autonomic nervous system activity (Shaffer and Ginsberg, 2017). Standard deviation of RR intervals (SDNN) was measured for HRV, since it is an index of timing variability associated with parasympathetic arousal. Shorter time between peaks of heart waves indicates decreased HRV, whereas longer time between peaks of heart waves represents increased HRV (Quintana and Heathers, 2014). In performance settings, HRV regulation induced by breathing may effective at impacting motor performance parameters essential in various sports (Jimenez Morgan and Molina Mora, 2017).

### Performance outcomes and data processing

Force data were processed offline using a customized LabVIEW program (14.0.1). Dependent measures included Absolute Error (AE), Variability (V), and Reaction Time (RT). RT was calculated by measuring the time between picture offset and the onset of force production. Force onset was measured as the time point when force production increased over a threshold of 6 Newtons force output produced during the 100 ms prior to image offset. RTs less than 100 ms following picture offset were deemed “anticipation trials” and removed by macros in excel and visual inspection. Additionally, trials with RTs or raw error scores ±3 standard deviations from the mean were removed. AE was calculated by subtracting each trial’s peak force from the target force value, squaring the difference, calculating the mean of the squared differences, then computing the square root of each mean value. AE scores represented total error on the task controlling for error direction (above or below the target). V was calculated by detrending each trial (force onset to peak force) and dividing the standard deviation of the detrended data by the mean force value. Seventy-one RT, forty-eight V, and seventy-three AE outlier trials were removed. Following exclusions, 89.63% of all trials were included in the analyses. Change scores were calculated for each dependent variable by taking the difference in the raw score in each breathing condition from the baseline condition.

### Instrumentation and task

**Figure 1:**
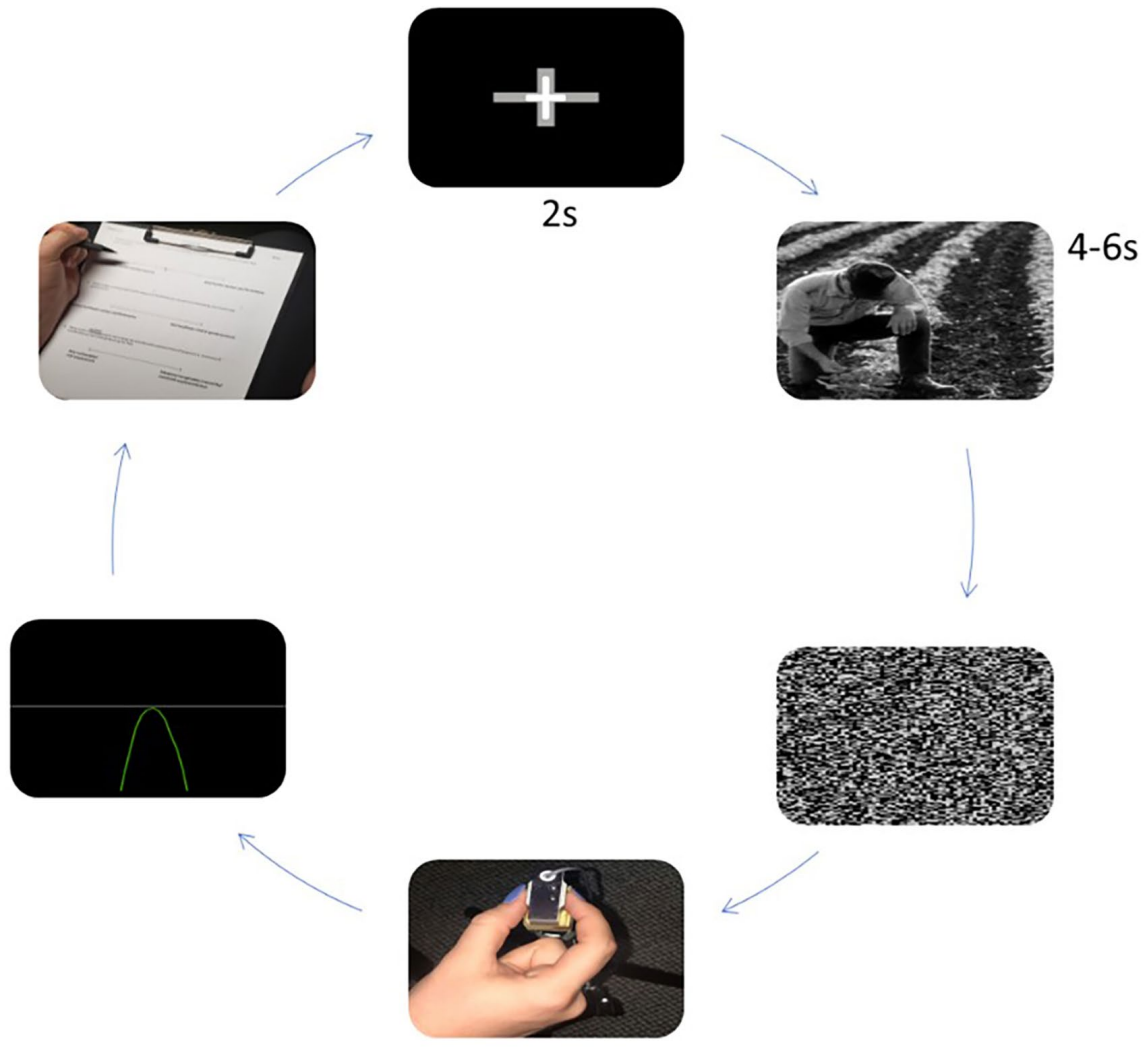
Set-up of experimental procedure.

Participants sat in a stationary chair with their head positioned 1 m from a computer screen (23.6 in, 60 cm; 1920 × 1,080 resolution) and performed isometric ballistic contractions with their right hand by pinching a force transducer (MLP-75, Transducer Techniques, Temecula, CA, United States) with the thumb and index finger (Figure 1). The force transducer’s vertical position was adjusted to allow participants’ right arms to relax comfortably on the arm rest at a right angle. Analog output from the force transducer was amplified through a 15LT Grass Technologies Physiodata Amplifier System (Astro-Med Inc. West Warwick, RI, United States) at an excitation voltage of 10 V. Custom LabVIEW software (14.0.1; National Instruments, Austin, TX) controlled trial onset, trial offset, visual stimulus presentation, and a 16-bit analog-to digital converter (A/D; PCI-6220, National Instruments, Austin, TX) which sampled the force at 100 Hz. Force data were analyzed offline. Participants’ breathing frequency was recorded by a force transducer belt (Pneumotrace; Morro Bay, CA, United States) placed 2 cm above their umbilicus around their abdomen. Participants were instructed to limit extraneous movement to reduce signal noise. In addition to the force transducer belt, participants wore a Polar heart rate monitor belt H10 around their chest (in line with xiphoid process). Measures were recorded during each breathing condition. We instructed participants to look at a fixation cross on the screen, and informed them that the fixation cross would eventually be replaced by an image. They were then told to maintain their attention on the image for the entire time it was presented. They were told to produce a force quickly and accurately as possible, equal to 10% of their maximum when the image disappeared. They did not receive feedback on their performance and were instructed to continue breathing with the metronome and wait until the next trial begins.

### Breathing manipulation

Participants performed slow-paced, normal-paced, or fast-paced breathing frequencies, each of which were paced by a metronome. Normal-paced breathing involved breathing at the same breaths per minute that were established during a baseline session (as described in the Experimental Design and Procedure section below). Slow-paced breathing involved altering breathing frequency to the pace exhibiting the largest increase in HRV, based on a previous protocol by Lehrer (Lehrer et al., 2000). Fast-paced breathing was set at an interval equivalent to the breaths per minute difference between slow- and normal-paced breathing. Prior to the current experiment, pilot testing of the fast breathing frequency was performed to ensure a fast frequency, but one that would safely avoid induction of hyperventilation. Breathing conditions were randomized and counterbalanced. Participants were instructed to breathe in and out with the metronome. They had approximately 1 min to acclimate to the breathing frequency prior to beginning the experimental condition. Participants were told to breathe in through their nose, and out through their mouth to prevent dry mouth (Bai, 1991). Lastly, participants were told to breath abdominally to ensure measurements were recorded from the strain gauge belt.

### Emotion manipulation

Images are commonly used manipulations of emotional experiences. They are categorized by valence (pleasant or unpleasant) and arousal (intensity; Lang et al., 2008). In the current experiment, participants viewed 30 randomized neutral, pleasant, and unpleasant digitized photographs within each condition. Neutral and blank images (black screens) are commonly employed to serve as a comparison between the presence and absence of complex visual stimuli, respectively. Biases can be found for facial images based on attractiveness, gender, and age, so an equal number of young, adult, and elderly images were secured for males and females. Facial images were used for neutral images, as they are more representative of a social interaction than inanimate objects. Pictures were selected according to affective normative ratings (NIMH, CSEA; Lang et al., 2008) from the International Affective Picture System. Due to an insufficient number of IAPS neutral face stimuli, internet search engines (e.g., Google, Pixabay, Yahoo) were used to obtain additional neutral face images of similar quality and content to the IAPS images. Image similarity was controlled by including an equal number of male and female children, young adults, and older adults.

Thirty unpleasant attack and pleasant erotic images were selected with ratings of arousal of at least 4.5, which is the minimum arousal required for changes to be seen in HRV. All images were set to grayscale and matched for contrast and brightness using Serif PhotoPlus [Version X8; Serif (Europe) Ltd., 2016] picture editing program. Image complexity was also controlled by selecting images containing one person, while also cropping out any distracting image details. In the current experiment, the final slideshow for each condition contained 30 images of the randomized faces, and opposite-sex unpleasant and pleasant images.

### Experimental design and procedure

Upon arrival to the lab, participants were greeted and provided further information about the study. They were prompted and afforded the opportunity to ask questions. Once questions were addressed, they were instructed to sit in a chair and to complete the informed consent. To begin the experiment, individualized maximal voluntary contraction (MVC: in Newtons) values were calculated for each participant using a previously established protocol (Vaillancourt and Newell, 2003; Coombes et al., 2009). Participants were trained to achieve motor task competency during two automated pre-experimental training sessions, requiring participants to produce a pinch force equaling 10% of their individualized MVC. A precision pinch grip task was completed by the thumb and index finger; however, additional digits can contribute to the force output variability of the task. Accordingly, to control for extraneous joint and muscles movements, participants wore a custom designed restraining sleeve that secures the additional digits in a flexed position with a hole cut to allow free movement of the thumb and index finger. The training session concluded once participants were able to complete eight out of ten of the most recent consecutive trials within a range of ±20% of the individualized target force.

Following the training session, participants progressed through a baseline condition followed by three randomized and counterbalanced breathing frequency conditions. Each of the three breathing conditions consisted of 30 trials completed free from experimenter interaction in a sound-attenuated room. To control for postural positions, participants wore a strap secured around the bottom of the rib cage and chair. Prior to each of the breathing conditions, participants breathed with a metronome at the required pace for 2 min and then continued to breathe with the metronome while performing the 10 pinch task trials.

Experimental conditions consisted of the presentation of a fixation cross (2 s) upon a black background, image presentation (4–6 s), and completion of the targeted pinch task upon onset of image pixilation. The duration of image presentation was randomized across conditions. Images and sequence orders were randomized across experimental conditions. Following each trial within the training sessions, participants received visual performance feedback. Performance feedback was not provided during baseline and breathing conditions. Feedback consisted of a white horizontal line, representing the 10% target force, and a green or red force trace that represented the force produced (Figure 1).

### Statistical analyses

The SSPS Statistics 22.0 package was used to conduct statistical analyses. Manipulation checks were conducted *via* separate 3 (Breathing Condition; slow breathing, normal breathing, and fast breathing) x 3 (Image Valence; pleasant, unpleasant, and neutral) repeated measures analyses of variance (ANOVA) of HRV responses, perceived valence, and perceived arousal. Motor performance responses to breathing conditions under emotional contexts were determined *via* separate 3 (Breathing Condition; slow breathing, normal breathing, and fast breathing) x 3 (Image Valence; pleasant, unpleasant, and neutral) ANOVAs for force outcomes: AE, RT, and V, and for visual analog scales: attention, dyspnea, valence, and hindrance. For significant main effects, Bonferroni adjusted pairwise comparisons were implemented. If the sphericity assumption was violated, then Greenhouse–Geisser degrees of freedom corrections were applied with a probability value set at *p* < 0.05 for all analyses.

## Results

### Manipulation check

Participants’ ratings of emotional stimuli for valence and arousal for unpleasant (Valence: *M* = 3.10, *SD* = 1.54; Arousal: *M* = 5.30, *SD* = 2.43), pleasant (Valence: *M* = 6.57, *SD* = 1.61; Arousal: *M* = 5.69, *SD* = 2.32), and neutral stimuli (Valence: *M* = 5.41, *SD* = 1.43; Arousal: *M* = 3.48, *SD* = 2.07) were consistent with the affective normative ratings of similar images contained within the International Affective Picture System (IAPS; Lang et al., 2008).

A main effect of Valence was identified, *F* (2, 2,838) = 1875.836, *p* < 0.001, *ƞ_p_^2^* = 0.569. *Post hoc* analyses indicated that unpleasant stimuli were rated significantly less pleasant than neutral (*p* < 0.001) and pleasant (*p* < 0.001) stimuli. Pleasant stimuli were also rated significantly more pleasant than neutral stimuli (*p* < 0.001). Further, ratings of the intensity (arousal) of emotional stimuli also significantly differed *F* (2, 2,838) = 505.259, *p* < 0.001, *ƞ_p_^2^* = 0.263. Unpleasant and pleasant stimuli were rated significantly more arousing than neutral stimuli (*p* < 0.001), while pleasant stimuli were rated as more arousing than unpleasant stimuli (*p* < 0.001).

### Force outcomes

The influence of breathing on motor performance was tested under each emotional context (Table 3 for all values).

**Table 3:** M (*SD*) values for breathing conditions while viewing neutral, pleasant, and unpleasant stimuli.

Variable	Conditions			
	Baseline	Slow	Normal	Fast
*Neutral*				
AE (*N*)	8.66 (26.74)	19.33 (47.25)	12.68 (22.15)	18.91 (31.42)
V (%)	12.40 (7.35)	14.67 (10.43)	14.10 (13.02)	15.01 (13.91)
RT (ms)	590.74 (242.52)	703.11 (341.42)	603.33 (294.51)	594.30 (290.76)
SDNN (ms)	46.90 (32.86)	78.26 (47.37)	50.80 (34.82)	41.41 (26.62)
*Pleasant*				
AE (*N*)	10.49 (28.65)	23.35 (57.24)	16.93 (33.74)	18.50 (33.62)
V (%)	11.86 (5.68)	13.97 (9.09)	13.66 (10.23)	14.41 (12.57)
RT (ms)	602.79 (254.47)	685.61 (336.07)	595.04 (236.02)	593.36 (306.15)
SDNN (ms)	47.05 (37.17)	77.65 (50.03)	52.00 (38.01)	46.42 (33.75)
*Unpleasant*				
AE (*N*)	9.88 (30.76)	20.78 (54.98)	14.38 (27.87)	18.39 (36.32)
V (%)	12.17 (6.01)	14.27 (10.11)	12.96 (7.61)	13.21 (9.31)
RT (ms)	605.50 (247.42)	709.12 (298.90)	611.85 (239.34)	642.69 (322.11)
SDNN (ms)	46.28 (30.81)	74.34 (46.36)	49.49 (32.15)	46.05 (34.73)

Means (*M*) and standard deviations (*SD*) are presented for each dependent variable: Absolute endpoint error (AE) in Newtons (*N*), Trial-to-trial Variation of the Force (V) as a percentage of force (%), and Reaction Time (RT) in ms, and Standard Deviation of the NN intervals (SDNN) in ms.

**Figure 2:**
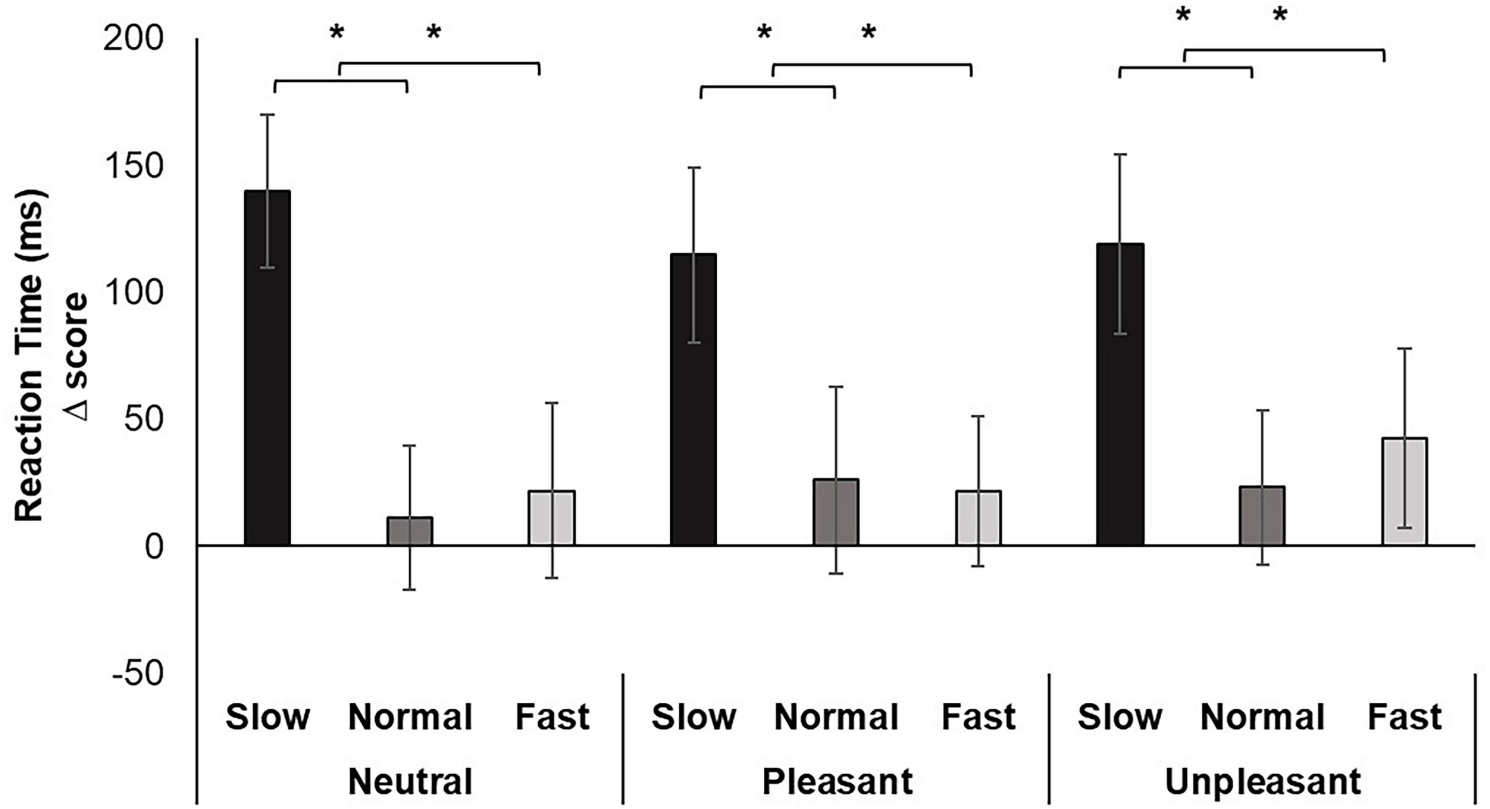
Mean RT change scores for each breathing frequency condition and image valence in milliseconds (ms). Greater values indicate slower reaction time. Error bars represent +1 SE from the mean. Asterisks represent significant differences (*p* < 0.05).

Repeated measures ANOVA revealed a significant main effect for breathing condition on reaction time, *F* (2, 74) = 19.463, *p* < 0.001, *ƞ_p_^2^* = 0.345. Pairwise comparisons indicated slow breathing significantly increased RT compared to normal and fast breathing conditions (*p*’s < 0.001). The effect of image valence, *F* (2, 74) = 0.098, *p* > 0.05, *ƞ_p_^2^* = 0.003, and the interaction of breathing and valence *F* (4, 148) = 1.061, *p* > 0.05, *ƞ_p_^2^* = 0.028 were nonsignificant (Figure 2).

**Figure 3:**
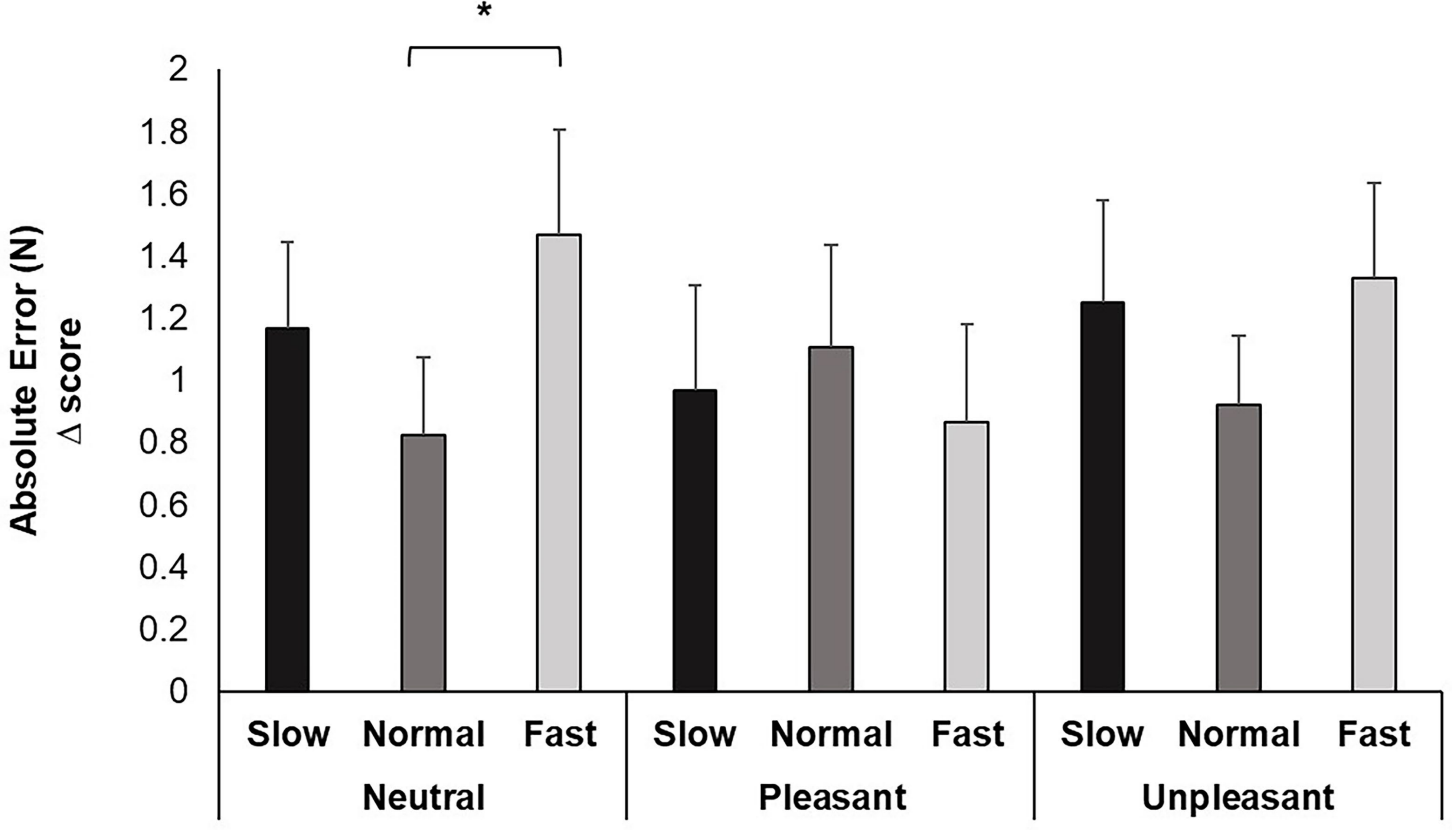
Mean AE change scores for each breathing frequency condition and image valence in milliseconds (ms). Greater values indicate less accuracy attaining target force. Error bars represent +1 SE from the mean. Asterisks represent significant differences (*p* < 0.05).

Repeated measures ANOVA identified a significant main effect of breathing condition for AE, *F* (2, 74) = 3.639, *p* < 0.05, *ƞ_p_^2^* = 0.168. Pairwise comparisons evidenced increased movement error when participants breathed fast compared to normal (*p* < 0.05). Although the image valence, *F* (2, 74) = 0.210, *p* > 0.05, *ƞ_p_^2^* = 0.012 and interaction of breathing and image valence, *F* (2, 74) = 1.545, *p* > 0.05, *ƞ_p_^2^* = 0.154 were nonsignificant, pairwise comparisons for the interaction revealed increases in error from fast breathing compared to normal occurred while viewing neutral images (*p* < 0.05; Cohen’s *f* = 0.43; Figure 3).

Repeated measures ANOVA yielded no significant effects of breathing condition on V, *F* (2, 74) = 1.579, *p* > 0.05, *ƞ_p_^2^* = 0.081, image valence, *F* (2, 74) = 1.856, *p* > 0.05, *ƞ_p_^2^* = 0.093, or the interaction between breathing and valence, *F* (4, 148) = 1.061, *p* > 0.05, *ƞ_p_^2^* = 0.111.

### Attention allocation, HRV, perceived arousal, hindrance, and dyspnea

See Table 2 for all values.

Change scores for attention allocation did not significantly differ among breathing conditions *F* (2, 60) = 1.728, *p* > 0.05, *ƞ_p_^2^* = 0.054.

**Figure 4:**
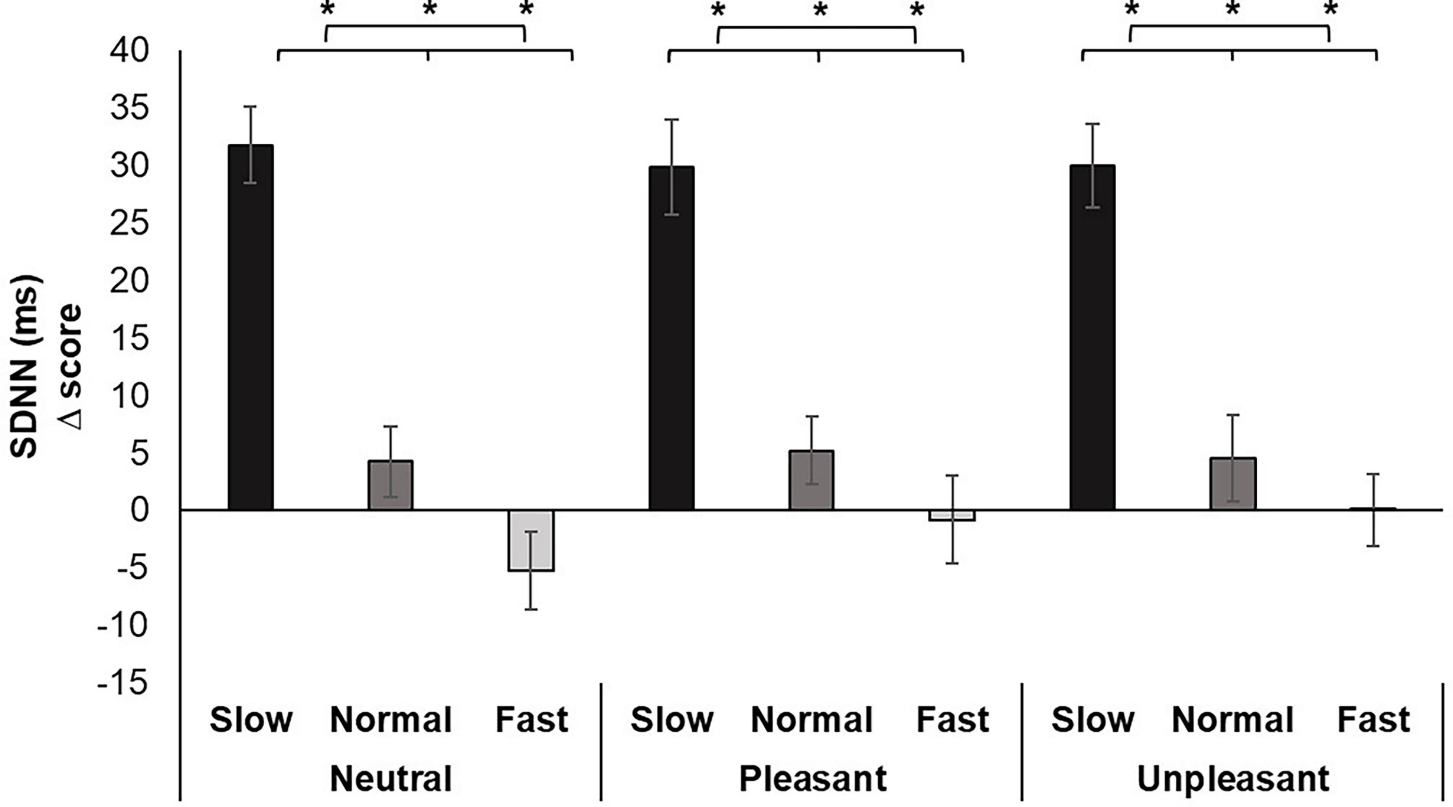
Mean SDNN change scores for each breathing frequency condition and image valence in milliseconds (ms). Greater values indicate increases in HRV. Error bars represent +1 SE from the mean. Asterisks represent significant differences (*p* < 0.05).

For HRV, a significant main effect was identified for Breathing condition *F* (2, 70) = 70.330, *p* < 0.001, *ƞ_p_^2^* = 0.668. Pairwise comparisons demonstrated slow breathing increased SDNN compared to normal and fast breathing (*p*’s < 0.001). In addition, similar increases from slow breathing were seen for all image valences (*p*’s < 0.01). Conversely, fast breathing significantly decreased SDNN compared to slow and normal breathing regardless of image valence (*p*’s < 0.05; Figure 4).

**Figure 5:**
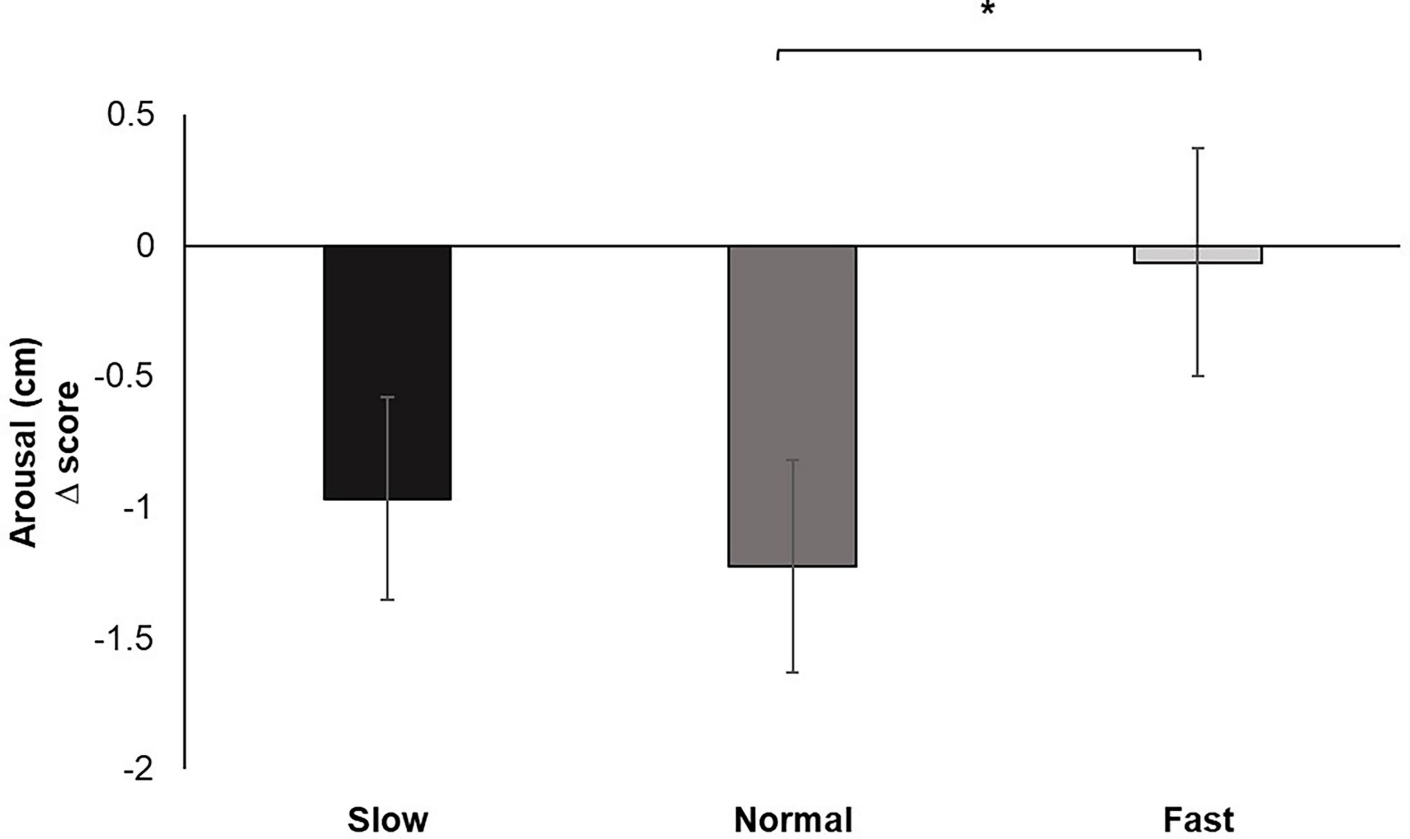
Mean perceived arousal change scores for each breathing frequency condition on a Likert scale from 1 to 9. Greater values indicate increases in perceived dyspnea of breathing to performance of the pinch task. Error bars represent +1 SE from the mean. Asterisks represent significant differences (*p* < 0.05).

Analysis of the change scores for Perceived Arousal identified a significant main effect for breathing condition, *F* (2, 60) = 4.407, *p* < 0.05, *ƞ_p_^2^* = 0.128. Pairwise comparisons demonstrated increased perceived ratings of arousal for fast breathing compared to normal (*p* < 0.05) breathing. Fast breathing did not significantly differ from slow breathing, nor did slow breathing significantly differ from normal breathing (Figure 5).

**Figure 6:**
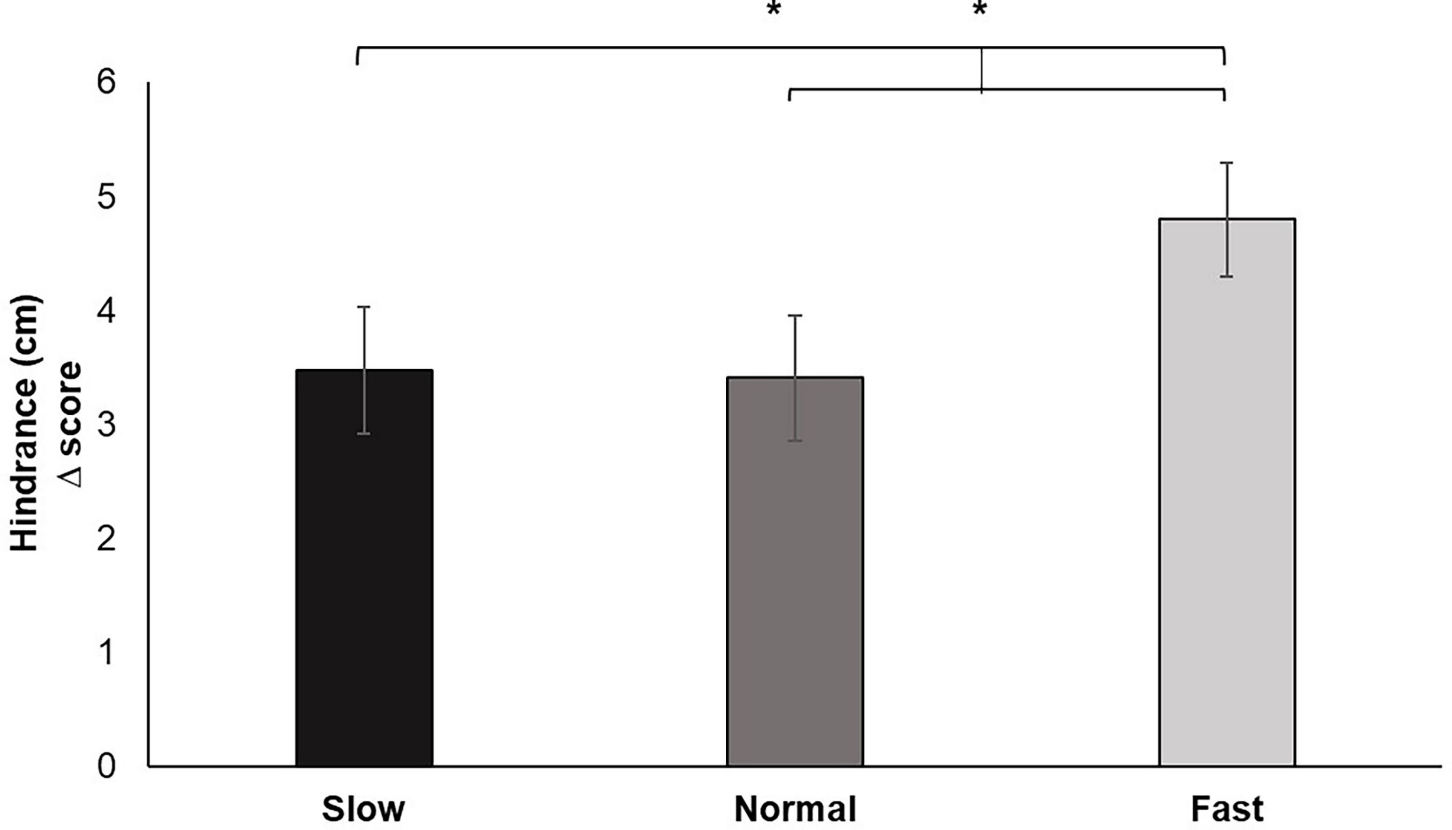
Hindrance change scores for each breathing frequency condition in centimeters (cm) on a scale from 1 to 10. Greater values indicate increases in perceived hindrance of breathing to performance of the pinch task. Error bars represent +1 SE from the mean. Asterisks represent significant differences (*p* < 0.05).

Analysis of the change scores for Hindrance (of breathing on pinch task performance) yielded a significant main effect for breathing condition, *F* (2, 60) = 5.668, *p* < 0.01, *ƞ_p_^2^* = 0.159. Pairwise comparisons confirmed increased ratings of hindrance under fast breathing compared to slow (*p* < 0.05) and normal (*p* < 0.05) breathing. Perceived hindrance for slow breathing did not significantly differ from normal (Figure 6).

**Figure 7:**
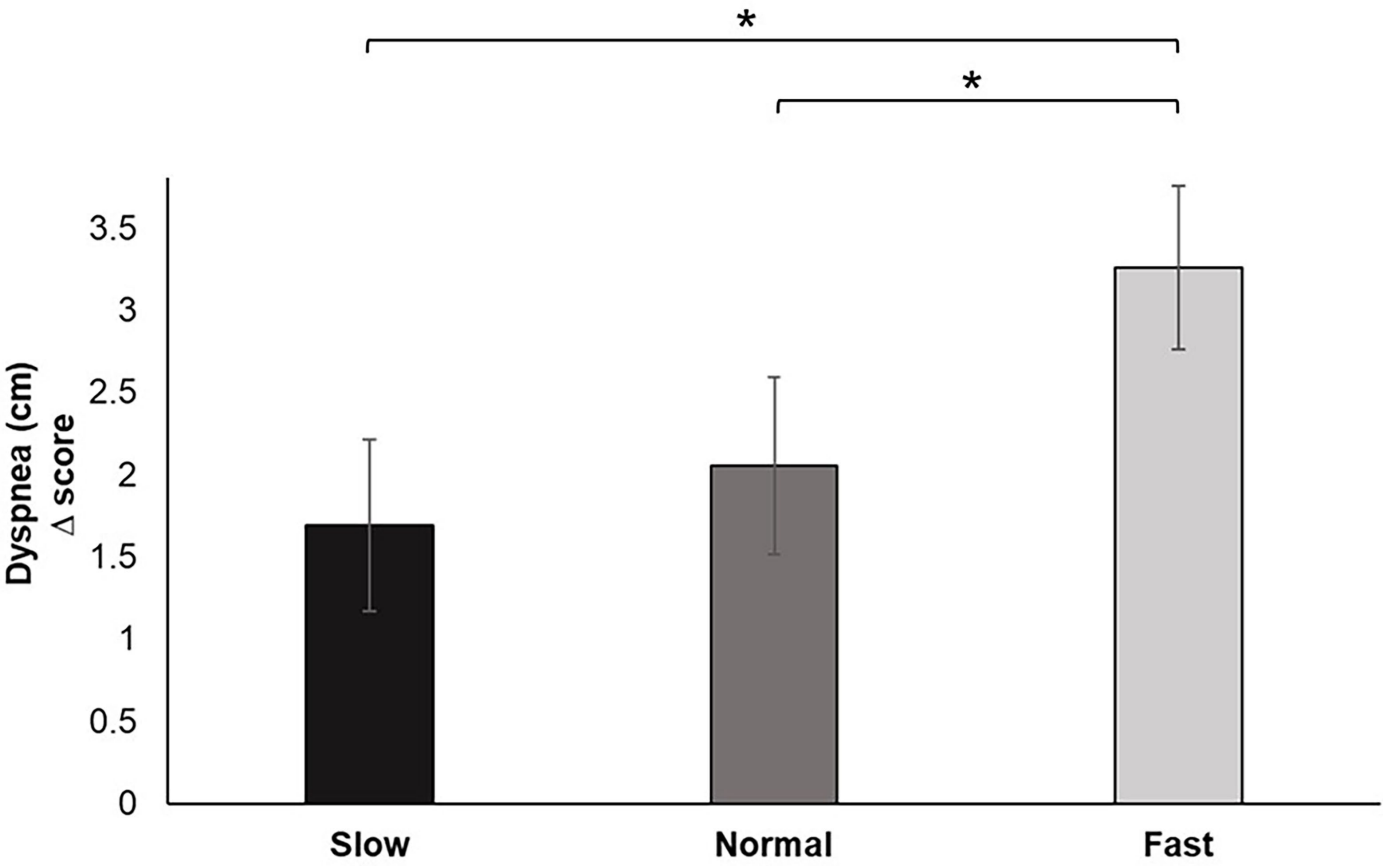
Dyspnea change scores for each breathing frequency condition in centimeters (cm) on a scale from 1–10. Greater values indicate increases in perceived dyspnea of breathing to performance of the pinch task. Error bars represent +1 SE from the mean. Asterisks represent significant differences (*p* < 0.05).

Analysis of the change scores for Dyspnea identified a significant main effect for breathing condition, *F* (2, 60) = 5.725, *p* < 0.01, *ƞ_p_^2^* = 0.160. Pairwise comparisons revealed that fast breathing increased feelings of perceived dyspnea during the pinch task performance compared to slow (*p* < 0.05) and normal (*p* < 0.01) breathing. Dyspnea reported during slow breathing did not significantly differ from normal (Figure 7).

### Hierarchical regression

Hierarchical multiple regression models showed that slower breathing frequency predicted increases in RT (*β* = −0.25, *p* < 0.05) for pleasant images, while predicting increases in HRV for unpleasant images (*β* = −0.45, *p* < 0.001). Increases in dyspnea (*β* = 0.29, *p* < 0.05) and hindrance (*β* = 0.35, *p* < 0.01) predicted increases in RT for pleasant images, while only increases in hindrance predicted increases in RT for unpleasant images (*β* = 0.41, *p* < 0.01). Slower breathing frequency predicted increases in HRV while viewing unpleasant images (*β* = −0.45, *p* < 0.001; Table 4).

**Table 4:** Hierarchical Regression Model Predictors of Performance Under Emotional Conditions.

Variable	Standardized *β* coefficient	*p-*value
Pleasant images		
*Reaction time*		
Breathing	−0.25	0.018
Dyspnea	0.29	0.030
Hindrance	0.35	0.004
Unpleasant images		
*Heart rate variability*		
Breathing	−0.45	0.001
*Reaction time*		
Hindrance	0.41	0.001

## Discussion

The primary aim of this study was to determine how breathing impacts the motor performance mechanisms that underlie sport tasks when motor skills must be executed quickly and accurately under variable emotional conditions. To accomplish this aim, participants engaged in various frequencies of metronome-paced breathing while viewing pleasant, unpleasant, and neutral images and performing a force pulse task. We hypothesized breathing frequency would predict changes in arousal under pleasant and unpleasant emotional conditions. We also expected increased attention to breathing would predict changes in variability and error under pleasant and unpleasant emotional conditions. Several novel findings emerged: (1) Slow breathing inhibited movement initiation, (2) Fast breathing disrupted movement accuracy within neutral contexts, (3) Perceptions of dyspnea and hindrance were greater when breathing fast, and (4) HRV was greatest when breathing slowly, regardless of emotional conditions. These novel findings are elaborated in the ensuing discussion, followed by acknowledgement of study limitations. Solutions are offered to address these limitations, and future research directions are recommended.

We hypothesized fast breathing rates would maintain reaction time compared to slow and normal breathing rates under pleasant and unpleasant emotional conditions. Prior research has established that fast breathing facilitates rapid execution of upper extremity movements. Previous work using a pinch initiation task like that used herein has shown decreased reaction times while breathing at an uncomfortable fast pace in conditions void of external emotional cues (Buchanan and Janelle, 2021). In the current study, participants breathed at various paces while also experiencing various emotional stimuli. The current results are not fully congruent with previous work. Fast breathing - although not hindering to reaction time - did not facilitate it either, raising questions of why the discrepancy in these findings. Exposure to unpleasant emotional content while performing regulation strategies requires increased attentional resources (Yuan et al., 2012). Our previous work focused on changes in fast breathing, yet we found that slow breathing had a comparatively greater impact on inhibiting movement initiation. Partially consistent with our hypothesis, reaction time was slower regardless of the affective context experienced when reducing breathing rate. In turn, the typical facilitation of expedited reactions occasioned under emotionally evocative circumstances did not occur. This reversal effect is consistent with breath regulation research indicating that slow breathing is an effective strategy for the purpose of emotion regulation, but delays reaction time of planning-dependent movements (Buchanan and Janelle, 2021). The delayed reaction time may also indicate that slow breathing disrupts the attentional process needed to detect task cues (i.e., image offset) and quickly initiate the motor response. An alternative explanation is that attention was drawn internally and away from the task. More time was present between slow breaths that could have distracted attention to the pinch task. However, while not significant, VAS scores for attention indicate participants were more attentive to the pinch task compared to fast breathing. Collectively, these findings suggest slow breathing downregulated all emotional experience.

We also hypothesized fast breathing rates would increase error compared to slow and normal breathing rates under pleasant and unpleasant emotional conditions. Under pleasant and unpleasant contexts motor accuracy was not impacted by the breathing task. However, under neutral contexts, fast breathing decreased motor accuracy. Active regulation of breathing, regardless of the pace, may have buffered the influence of emotional experiences on motor action. Previous work suggests that more challenging regulation strategies elicit increases in analogs of absolute error (Beatty et al., 2014). In the current study, fast breathing increased error compared to slow and normal breathing conditions only under neutral conditions. Generally, overshoot errors are more common (i.e., greater force production) under highly arousing emotional experiences (Coombes et al., 2009). With regard to executing a memory guided force pulse in particular, participants in our prior studies have reported greater arousal, while also overshooting the target force of 10% MVC (Buchanan and Janelle, 2021). This finding was replicated in the current study, while expanding on associations of error with other performance outcomes. Despite these context-specific connotations, our results suggest that attending to emotional stimuli buffer expeditious effects of increased respiratory rate.

Perceptions of dyspnea and hindrance were greater when breathing fast. While arousal and valence are integral to impacts on motor performance, attentional mechanisms contribute to deviations in motor parameters as well. Further, associations exist between indices of arousal and attention. Arousal and attention have entangled associations with breathing rate. Physiological arousal changes in HRV can be influenced by manipulating breathing rate. Manipulating breathing also causes increased workload on the respiratory system (dyspnea) and may impact the sense of task competence (hindrance). Therefore, measures of physiological arousal (HRV), perceived arousal, dyspnea and hindrance index attention to a goal-directed task. Overall, results partially support our hypothesis that emotion and attention contribute to the effects of breathing rate on motor performance. Relative to slow and normal breathing, fast breathing increased feelings of dyspnea and hindrance during task performance. Supported by regression findings in the current study, self-reported feelings of discomfort were associated with increased reaction time.

Slow breathing increased HRV compared to fast and normal breathing across all emotional conditions. Otherwise stated, slow breathing reduced physiological arousal. The consequent reduction in physiological arousal evidently contributed to delays in response time. This assertion is consistent with the identified strong negative correlation between response time and various indices of physiological and perceived arousal. Reported attention was allocated more to the pinch task than the breathing task, but, contrary to our hypothesis, the degree to which attention was allocated did not differ between breathing conditions. As highlighted in Thayer and Lane’s Neurovisceral Integration Model, a regulatory link exists between emotions and HRV activity *via* mediation of the vagus nerve, which is responsible for the autonomic feedback loop detected by the baroreceptors (Thayer and Lane, 2000). Baroreceptor activity is constantly detecting changes in cardiovascular activity such as, heart rate (Holwerda et al., 2016). Heart rate increases with inhalations and decreases with exhalations and this synchronistic activity is termed respiratory sinus arrhythmia (RSA; Shader et al., 2018). RSA and breathing directly influence HRV, or physiological arousal (Topçu et al., 2018). Under neutral conditions, fast breathing increased error compared to slow breathing. The influence of slow breathing on arousal *via* autonomic regulation gives credence to the use of breathing interventions in performance settings.

The current investigation provided the opportunity to explore potential psychophysiological mechanisms that may underlie the alterations in motor performance that emerged under varying breathing conditions. Findings suggest perceived and physiological mechanisms of emotion contribute to breath regulation induced changes in motor performance. However, several limitations of the current study should be noted. First, the study was conducted with a sample of healthy young adult participants so should be cautiously interpreted in terms of implications for athletes and sport performance. In addition to the dimensional aspects of emotion, importance was placed on selection of measurement instruments in determining the impact of emotion on behavior. Different emotional response systems exist, and measurement of those response systems has limitations. Therefore, the need remains to integrate and triangulate these measures to the greatest extent possible. Task limitations of ecological validity and generalization to gross motor tasks also need to be acknowledged, based on our use of a fine motor task. Limitations acknowledged, the findings reported herein hold strong potential for advancing our understanding of how breathing interventions can be tailored to address the unique emotion regulation and motor requirements of specific sports and sport task components. In the context of the TIMER model, implementing breath regulation as an antecedent emotion regulation strategy is dependent on the emotional contexts and task goals (Beatty and Janelle, 2020). Our data highlight slow breathing created a greater consequence to movement initiation compared to fast breathing. Thus, fast breathing is most ideal in environments where speed is emphasized, such as tennis, which includes short bursts of needed speed, or in football, where a running back needs to quickly move the ball forward. Slow breathing is most appropriate when accuracy must be maintained under minimal time constraints, such as archery or golf (Jimenez Morgan and Molina Mora, 2017). With replication and extension of these findings, sport psychology consultants will be able to confidently recommend the type of breathing approach based on our recommendations, as informed by the time periods outlined in the TIMER model. We hope to see breathing interventions further taught and customized to athletes as part of their practices, and to assist with overall autonomic regulation.

In conclusion, our findings advance the literature demonstrating arousal impacts motor efficiency, while feelings of dyspnea and hindrance affect motor efficacy. Additional work is needed concerning how breathing interventions broadly, and manipulations of breathing rate in particular, impact motor planning and execution under varying emotional contexts. Specifically, future work should investigate whether more objective measures of attention are related to breathing-induced alterations in performance. Future studies examining the effectiveness of manipulating breathing frequency to regulate both perceived and physiological dimensions of emotional responses as well performance enhancement remain ripe for empirical inquiry.

## Data availability statement

The raw data supporting the conclusions of this article will be made available by the authors, without undue reservation.

## Ethics statement

The studies involving human participants were reviewed and approved by University of Florida Institutional Review Board. The patients/participants provided their written informed consent to participate in this study.

## Author contributions

All authors listed have made a substantial, direct, and intellectual contribution to the work, and approved it for publication.

## Funding

This research was supported by the North American Society for the Psychology of Sport and Physical Activity (NASPSPA), Graduate Student Research Grant.

## Conflict of interest

The authors declare that the research was conducted in the absence of any commercial or financial relationships that could be construed as a potential conflict of interest.

## Publisher’s note

All claims expressed in this article are solely those of the authors and do not necessarily represent those of their affiliated organizations, or those of the publisher, the editors and the reviewers. Any product that may be evaluated in this article, or claim that may be made by its manufacturer, is not guaranteed or endorsed by the publisher.
